# Species‐Specific Response of Fish Health Condition to Nutrient Enrichment in Subtropical Rivers

**DOI:** 10.1002/ece3.73353

**Published:** 2026-04-02

**Authors:** Jianwen Li, Zhenmei Lin, Jinlei Yu, Zhigang Mao, Shi Fu, Kun Xu, Chan Li, Junfeng Gao, Kuanyi Li, Zhengwen Liu

**Affiliations:** ^1^ State Key Laboratory of Lake and Watershed Science for Water Security, Nanjing Institute of Geography and Limnology Chinese Academy of Sciences Nanjing China; ^2^ University of Chinese Academy of Sciences Beijing China; ^3^ Sino‐Danish Centre for Education and Research University of Chinese Academy of Sciences Beijing China; ^4^ Department of Ecology Jinan University Guangzhou China

**Keywords:** condition factor, eutrophication, Lake Chaohu Basin, length—weight relationships, polder

## Abstract

Fish growth is closely related to environmental variables; however, how nutrient enrichment affects fish growth conditions remains unclear in subtropical polder rivers. We investigated the growth status of six fish species and nutrient concentrations at 40 sites in the subtropical polder rivers of the Lake Chaohu Basin, with the aim of evaluating how the fish condition factor (*K*) responds to nutrient enrichment across different feeding groups. We observed species‐specific responses of growth conditions to nutrient enrichment. The omni‐zooplanktivorous *Hemiculter leucisculus* and zooplanktivorous *Toxabramis swinhonis* exhibit better growth in nutrient‐enriched rivers, with their *K* values showing a significant positive correlation with both total nitrogen (TN) and total phosphorus concentrations. However, the *K* values of the omni‐benthivorous *Carassius gibelio* and *Pseudobrama simoni* exhibited no significant correlation with nutrient enrichment. Interestingly, the condition factor of the piscivorous *Culter* species also increased with rising TN levels. The growth patterns of *H. leucisculus* and *C. gibelio* were positive allometric, where the fish body weight increased at a faster rate than length. *T. swinhonis* and *P. simoni* showed isometric growth, in which both the body weight and length of them increased at approximately the same rates. These findings highlight the importance of considering fish functional traits (e.g., feeding guilds) when assessing the ecological impacts of eutrophication and provide insights for the management of subtropical polder river ecosystems under nutrient enrichment, such as predicting changes in fish community structure and developing targeted conservation strategies.

## Graphical abstract

Fish condition factor (*K*) can be used to evaluate the effects of eutrophication.

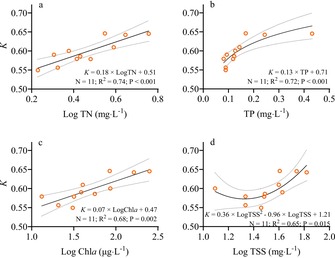

Species‐specific responses of fish K to nutrient enrichment were detected.

Nutrient enrichment significantly enhanced the *K* value of zooplanktivorous fish, while it did not affect the *K* value of omnivorous fish.

## 1 Introduction

Fish constitute a crucial component of aquatic ecosystems, and their growth is closely correlated with environmental variables. Length—Weight Relationships (LWRs) and its derived indices are widely employed to assess fish growth status (Konoyima et al. 2020; Karimov et al. 2024; Mondal et al. 2024). In the LWRs of fish, the *b* represents the allometric factor, which reflects the growth rate of the fish (Keys 1928). Therefore, *b* is a useful indicator to assess the fish growth patterns among different living environments. In addition, Fulton's condition factor (*K*) is commonly used to evaluate the fish health conditions in fish biology and aquaculture studies (Fulton 1904; Ighwela et al. 2011; Giosa et al. 2014). The value of *K* < 1 indicates that fish grows in a unfavorable habitat, whereas *K* > 1 indicates a favorable environment; therefore, the higher *K* value represents the better health condition (Lecren 1951; Safran 1992; Petrakis and Stergiou 1995; Froese 2006). However, how *K* responds to environmental changes in fish with different feeding habitats remains unclear (Perry et al. 2005; Cheung et al. 2013).

Eutrophication, caused by nutrient enrichment (e.g., nitrogen and phosphorus), induces substantial alterations in freshwater ecosystems (Carpenter et al. 1998; Smith 2003), including the proliferation of phytoplankton and the decline in water transparency (Jeppesen et al. 2007). These changes may affect fish health by influencing their food availability or predation efficiency (Hessen et al. 2006; Wang et al. 2019). Previous studies have shown that the *K* values of various fish species are closely related to nutrient levels. For example, the *K* value of *Saurogobio dabryi* in the Jialing River was negatively correlated with the concentration of ammonia nitrogen (${\mathrm{NH}}_{4}^{+}$) (He 2023). However, in the Yenisei River, the *K* value of *Thymallus arcticus* showed positive correlations with chlorophyll‐*a* (Chl*a*) and total phosphorus (TP) concentrations (Zuev et al. 2017). Furthermore, in northern Vietnam, *Bostrychus sinensis* exhibited higher *K* values during the rainy season, which coincided with the increase in both Chl*a* and TP (Nguyen et al. 2023). The contradictory findings on how fish health responds to environmental variables may reflect interspecific differences, which are often associated with variations in the feeding habits of different species. Because the potential prey resources of fish respond in a species‐specific manner to nutrient enrichment, for example, in Lake Taihu, the biomass of both zooplankton and macroinvertebrates increases alongside phytoplankton biomass (Cai et al. 2011; Jia et al. 2017), however, the coverage of submerged macrophytes decreased with TP in 71 Danish lakes (Jeppesen et al. 2000). Therefore, the health status of fish dependent on these food sources could be indirectly impacted by the increased nutrient levels.

In our study, we investigated the Length—Weight Relationships (LWRs) and condition factors (*K*) of six fish species, along with the water quality variables of their habitats, to explore how the growth status of fish responds to nutrient enrichment across 40 sampling sites in the subtropical polder rivers within the Lake Chaohu Basin. We hypothesized that the *K* value of zooplanktivorous and omnivorous fish would improve with nutrient enrichment, as enriched nutrients increase the availability of their prey. Conversely, the *K* value of piscivorous fish is predicted to decline with nutrient enrichment, since eutrophication‐induced reductions in water transparency impede their visual foraging efficiency (Miner and Stein 1996; Jonsson et al. 2013; Li et al. 2013).

## 2 Materials and Methods

### 2.1 Study Sites

**FIGURE 1:**
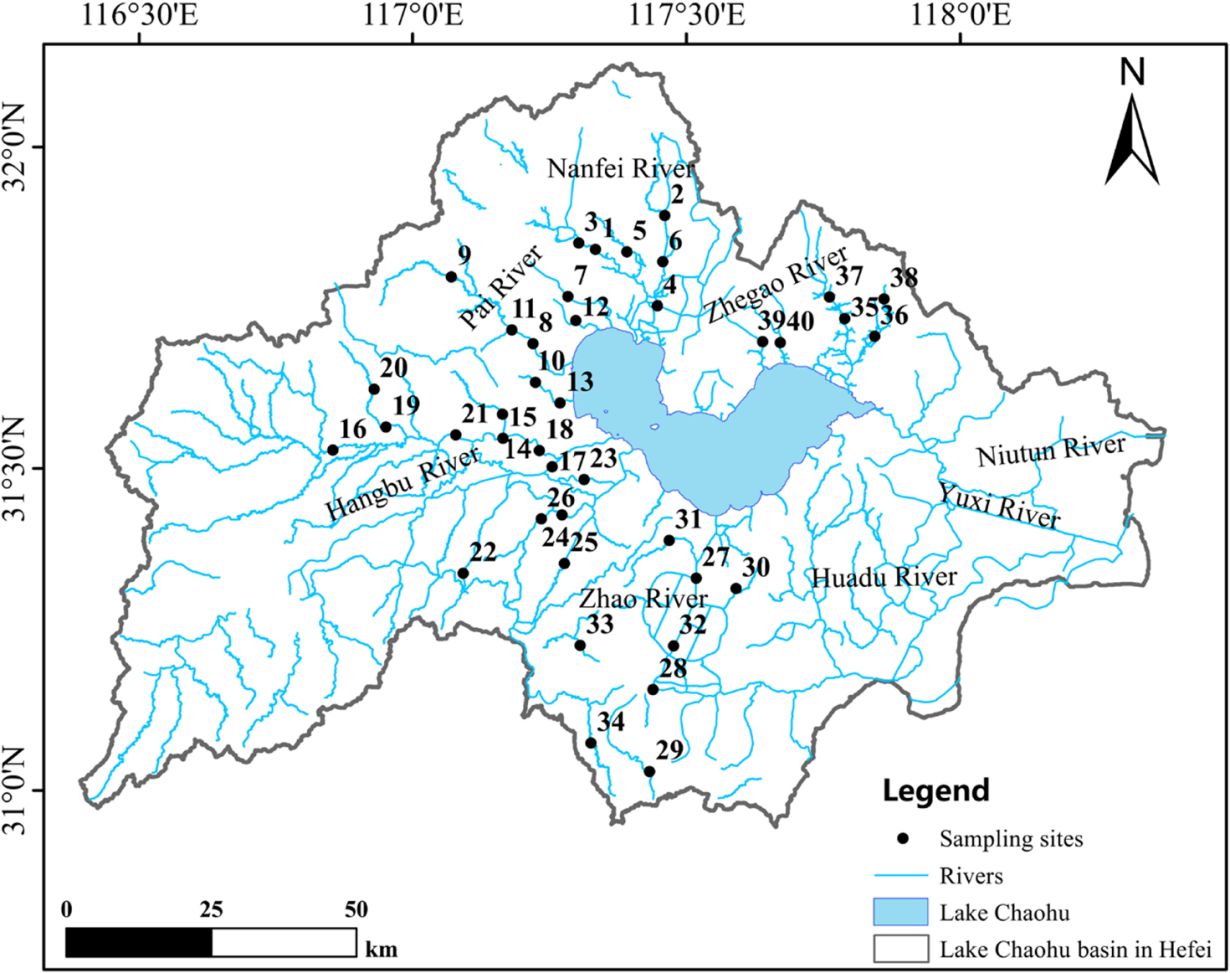
Distribution of the sampling sites for fish and water quality in rivers of Lake Chaohu Basin in Hefei, Anhui Province.

Lake Chaohu Basin (30°50′ – 32°10′ N, 116°20′ – 118°25′ E) is located in the middle and lower reaches of Yangtze River, in Anhui Province, covering an area of 1.41 × 10^4^ km^2^ (Figure 1). This basin is marked by intensive agricultural and urban activities, which result in elevated nutrient inputs and recurrent algal blooms, rendering it one of the most eutrophic large lake basins in China (Wang et al. 2020; Yang et al. 2020). In July 2023, we collected fish and water samples from 40 sampling sites situated in the subtropical polder rivers within the Lake Chaohu Basin (Figure 1, Table S1). The mean straight‐line distance between adjacent sampling sites on the same river was 7.5 km (with a range of 3.1–13.8 km).

### 2.2 Sampling Collection and Analysis

Fish were collected using a multi‐mesh gill net composed of eight distinct mesh sizes (5, 10, 15, 20, 25, 30, 35, and 40 mm) (Yu et al. 2021). Each mesh‐sized gillnet was 10 long and 1.5 m wide. The gillnets were connected in ascending order of mesh size, forming a total length of 80 m. In July 2023, fish were collected once at each of the 40 sites using new multi‐mesh gillnets. After 2–3 h, the net was retrieved, and the fish caught in it were removed and identified to the species level. Total length (*L*) and wet body weight (*W*) were measured to the precision of 0.1 cm and 0.1 g, respectively, and feeding habits were classified into omni‐zooplanktivore, zooplanktivore, omni‐benthivore, and piscivore (Teixeira‐de Mello et al. 2009).

Water samples were collected from three different layers (0.5, 1.0, and 1.5 m) using a 5‐L glass sampler and then mixed in a plastic bucket. Subsamples (2.5 L) were transported to the laboratory for further analysis. Total nitrogen (TN) was measured using an alkaline potassium persulfate digestion followed by UV spectrophotometry. Total phosphorus (TP) was determined using the ammonium molybdate spectrophotometric method after digestion with K_2_S_2_O_8_. Chlorophyll‐*a* (Chl*a*) was extracted with 90% acetone (v/v) and measured by spectrophotometry without correction for pheophytin interference. Total suspended solids (TSS) were determined by filtering 0.5–2 L of water through pre‐weighed GF/C filters, followed by drying at 60°C. All the variables mentioned above were analyzed in accordance with the Chinese Standard Methods for Monitoring Lake Eutrophication (Jin and Tu 1990).

### 2.3 Data Analysis

Length—Weight Relationships (LWRs) of the fish were determined using all individuals of the same species collected from each sampling site, according to the following formula (Keys 1928):

$$W={\mathrm{aL}}^{b}$$

where *W* is the wet body weight (g), *L* is the total length (cm), *a* is the coefficient, and *b* is the allometric factor.

For each species at each site, the condition factor (*K*) was calculated individually for every captured fish using the following formula (Fulton 1904):

$$K=100\times \left(\frac{W}{L^{3}}\right)$$

where *W* is the wet body weight (g) and *L* is the total length (cm) of the target fish species.

In field sampling, a total of over 30 fish species were captured; however, only six species were selected for analysis, as they yielded sufficient specimens at each site. The selected species included omni‐zooplanktivorous *Hemiculter leucisculus* and zooplanktivorous *Toxabramis swinhonis* (Yu et al. 2016); omnivorous benthivores *Carassius gibelio* and *Pseudobrama simoni* (Zhang et al. 2020; Gu et al. 2021); and piscivorous *Culter mongolicus* and *Culter alburnus* (Qin et al. 2021; Wang et al. 2022). The criteria for filtering species were applied based on the abundance and occurrence of each species. The site abundances of *H. leucisculus* and *T. swinhonis* were relatively high, thus the number of individuals was over 10 in each selected site and the coefficient of determination (*R* ^*2*^) of the LWRs was above 0.9 (Froese 2006). For *C. gibelio* and *P. simoni* , the criterion was set at individuals over 3 and *R* ^*2*^ exceeding 0.9. For the piscivorous *Culter* species, which share similar feeding habits, growth patterns, and trophic niches (Chen et al. 2009; Wang et al. 2019, 2022), they were grouped as *Culter* spp. to ensure sufficient representation. The *K* and *b* calculations of *Culter* spp. were performed only when both species were captured at the same site and met the individuals over 3 and R^2^ exceeding 0.9 criteria.

### 2.4 Statistical Analysis

Spearman correlation analysis and Generalized Linear Models (GLMs) were performed on water quality variables to characterize their inherent coupling relationships. Water quality variables were log‐transformed (TN: log_10_ (x + 1); Chl*a* and TSS: log_10_ x). These transformations were to improve the satisfaction of the normality and homoscedasticity assumptions, thereby facilitating subsequent analysis.

The relationship between *K* and water quality variables were quantifying by Spearman correlation analysis. Subsequently, Generalized Additive Models (GAMs) were employed to explore potential relationships between *K* and water quality variables, leveraging their well‐known capacity to handle the non‐repeated measures commonly encountered in such contexts (Spiegel et al. 2019; Aeberhard et al. 2021). The assumptions of the GAMs were evaluated using residual plots and Q—Q plots to verify the normality, statistical independence, and homoscedasticity of the residuals. The adequacy of the smooth terms was verified using the *gam. check*() function from the “*mgcv*” package, ensuring sufficient basis function dimensions (*k*‐index > 1, *p* > 0.05) and no evidence of overfitting (Wood et al. 2016). Smooth terms with *k* = 3 performed adequately. To further clarify the relationships identified by the GAMs, we applied GLMs to assess which functional form (linear, logarithmic, quadratic, or exponential) best described the associations between *K* and the significant water quality variables. The relative performance of these models was evaluated using the Akaike Information Criterion (AIC), with lower values signaling superior model fit through an optimal balance of explanatory power and parsimony (Zuur et al. 2010). All statistical analyses were performed using *R* v4.4.1.

## 3 Results

### 3.1 Physicochemical Variables

In our sampling sites, the mean concentration of TN was 2.4 mg・L^−1^ (range 0.7–6.0 mg・L^−1^), TP was 0.15 mg・L^−1^ (range 0.06–0.43 mg・L^−1^), Chl*a* was 70.3 μg・L^−1^ (range 4.3–342.2 μg・L^−1^), and TSS was 31.5 mg・L^−1^ (range 11.6–84.0 mg・L^−1^).

**FIGURE 2:**
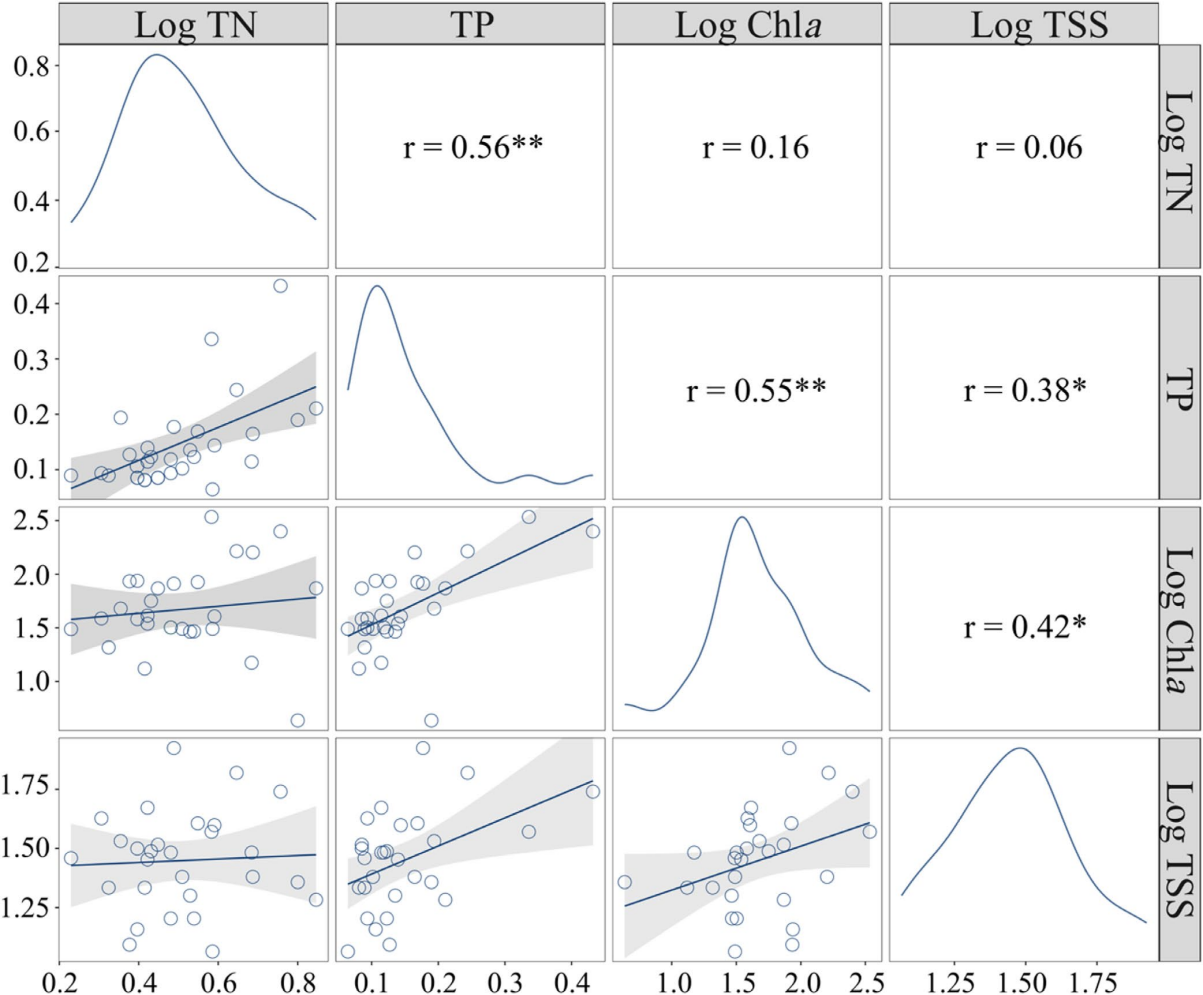
Relationship between water quality variables. The upper triangular matrix displays the corresponding Spearman correlation coefficients in numerical form. The lower triangular part uses scatter plots and Generalized Linear Models (GLMs) to display the relationships between water quality variables, where the solid line represents the fitted curve and the shaded area denotes the 95% confidence interval. Asterisks indicate the level of statistical significance, *p* < 0.05 (*) and *p* < 0.01 (**).

Spearman's correlation analysis revealed that TN was significantly positively correlated with TP, while TP showed significant positive correlations with both Chl*a* and TSS (Figure 2). Meanwhile, TSS and Chl*a* also showed positive correlation (Figure 2). Among these significant relationships, the strongest correlation was observed between TN and TP (*r* = 0.56, *p* = 0.002), followed by TP and Chl*a* (*r* = 0.55, *p* = 0.003). In contrast, the weakest correlation was found between TSS and TP (*r* = 0.38, *p* = 0.044).

### 3.2 Length—Weight Relationships (LWRs)

**FIGURE 3:**
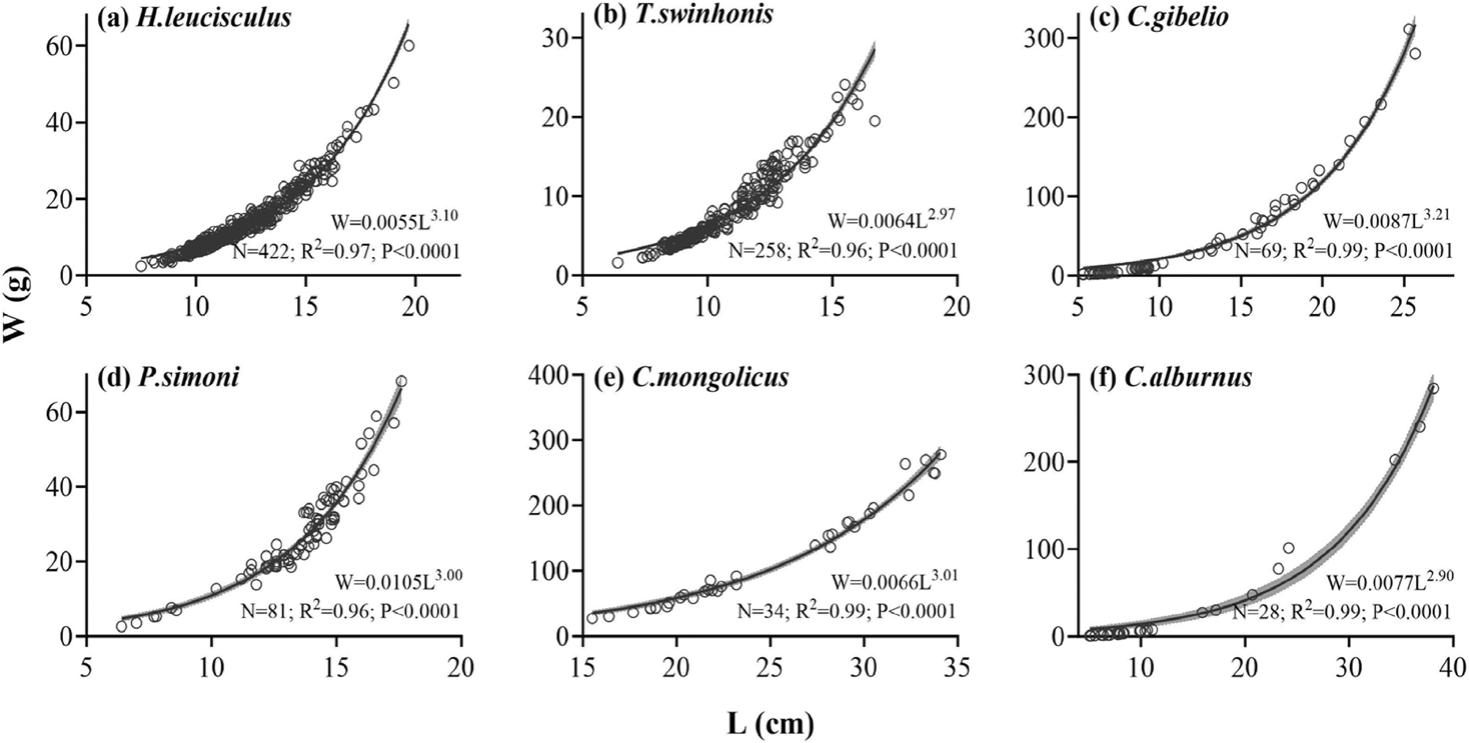
Length – Weight Relationships (LWRs) of six fish species. The relationship between total length (L, cm) and wet body weight (W, g) was modeled using power function fits; solid lines represent the fitted curves for each species, and the shaded area indicates the 95% confidence intervals. *N* denotes the number of individuals analyzed. The *R* ^*2*^ (coefficient of determination) and all *P* values indicate the goodness of fit and the overall significance of the LWRs models for the fitted curves, respectively.

The *b* values of both *H. leucisculus* and *C. gibelio* were significantly greater than 3, indicating positive allometric growth, where body weight increased at a faster rate than body length (Table 1). The *b* values of *T. swinhonis* , *P. simoni* , and *C. mongolicus* did not differ significantly from 3 (Table 1), indicating isometric growth, where the body weight and length of these species increased at approximately the same rate. However, the *b* value of *C. alburnus* was significantly lower than 3, indicating a negative allometric growth pattern in which body weight increases at a slower rate relative to body length (Table 1, Figure 3).

**TABLE 1:** Length—Weight relationships (LWRs) of six fish species.

Species	*N*	*L*_mean_ ± SD	*L*_range_ (cm)	*W*_mean_ ± SD	*W*_range_ (g)	*a*	*b*	*R* ^2^	*P*_*b* = 3_
*H. leucisculus*	422	12.1 ± 2.1	7.5–19.7	13.8 ± 8.0	2.4–60.0	0.006	3.10	0.97	< 0.0001
*T. swinhonis*	258	10.7 ± 1.9	6.4–16.7	8.1 ± 4.5	1.6–24.1	0.006	2.97	0.96	0.40
*C. gibelio*	69	11.8 ± 5.6	5.3–25.7	46.2 ± 66.2	1.9–311.1	0.009	3.21	0.99	< 0.0001
*P. simoni*	81	13.8 ± 2.4	6.4–20.4	28.7 ± 12.4	2.6–68.3	0.011	3.00	0.96	0.97
*C. mongolicus*	34	24.8 ± 5.7	15.5–34.1	121.1 ± 79.9	27.0–277.9	0.007	3.01	0.99	0.80
*C. alburnus*	28	13.2 ± 9.7	5.1–38.1	38.7 ± 76.5	0.9–284.3	0.008	2.90	0.99	0.01

*Note:* *N*, number of fish individuals; *L*_*mean*_, mean total length; *SD*, standard deviations; *L*_*range*_, ranges of total length; *W*_*mean*_, mean wet body weight; *W*_*range*_, range of wet body weight; *a*, coefficient; *b*, allometric factor; *R* ^*2*^, the coefficient of determination of the LWRs function; *P*_*b = 3*_, the statistical significance level of the difference between the estimated *b* value and 3.

### 3.3 Fish Condition Factor (*K*)

The mean *K* values of *H. leucisculus* , *T. swinhonis* , and *Culter* spp. were all below 1 in our study (Table 2). However, the mean *K* values of both *C. gibelio* and *P. simoni* were greater than 1 (Table 2).

**TABLE 2:** Condition factor of each fish species.

Species	*N*_sites_	*K*_*mean*_ ± SE	*K*_range_	*b*_*mean*_ ± SE	*b*_range_
*H. leucisculus*	19	0.70 ± 0.01	0.64–0.77	3.07 ± 0.04	2.76–3.41
*T. swinhonis*	11	0.60 ± 0.01	0.55–0.65	2.95 ± 0.09	2.57–3.42
*C. gibelio*	8	1.51 ± 0.04	1.28–1.66	3.16 ± 0.95	2.62–3.53
*P. simoni*	7	1.07 ± 0.04	0.95–1.21	3.19 ± 0.18	2.64–3.85
*Culter* spp.*	12	0.66 ± 0.01	0.58–0.76	3.11 ± 0.12	2.50–4.11

*Note:* *N*_*sites*_, total number of sampling sites where each species was recorded; *K*_*mean*_, mean condition factor; *K*_*range*_, range of condition factor; *b*_*mean*_, mean allometric factor; *b*_*range*_, range of allometric factor.

* For *Culter* spp., the *K* values were calculated by averaging the *K* values of *C. mongolicus* and *C. alburnus* at each sampling site, followed by determining the overall mean across all sites.

### 3.4 Relationship between Fish *K* and Water Quality Variables

**FIGURE 4:**
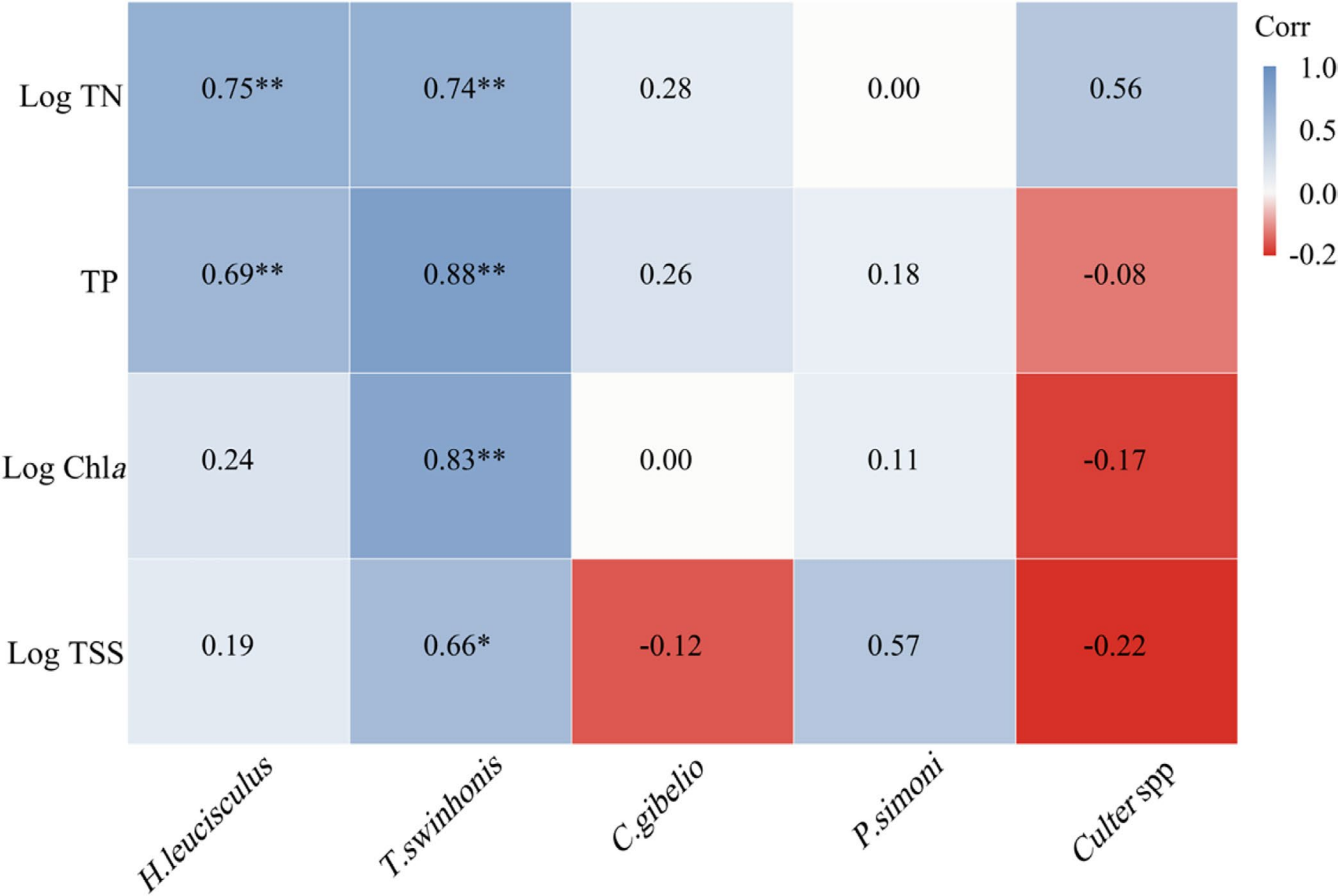
Spearman's correlations between fish condition factor (*K*) and water quality variables. The heatmap illustrates the correlation coefficients between the condition factor of fish species with different feeding habits and water quality variables. Correlation strength and direction are represented by color gradients. The *Culter* spp. includes both *C. mongolicus* and *C. alburnus* . Asterisks indicate levels of statistical significance: *P* < 0.05 (*), *p* < 0.01 (**).

Based on the correlation analysis results, we found that the *K* values of both *H. leucisculus* and *T. swinhonis* were significantly positively correlated with TN and TP, respectively (Figure 4). Moreover, *T. swinhonis* also showed a positive correlation with both Chl*a* and TSS concentrations (Figure 4). The *K* values of the two piscivorous *Culter* species showed a positive correlation with TN (*p* = 0.058). However, the *K* values of both *C. gibelio* and *P. simoni* were not significantly affected by these water quality variables (Figure 4).

**FIGURE 5:**
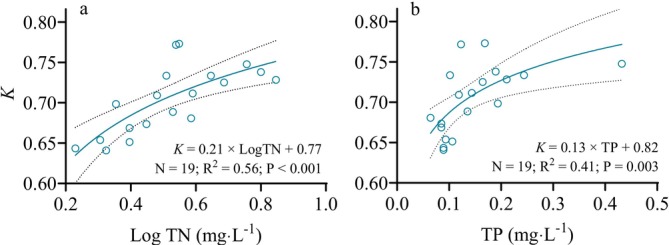
Relationship between the condition factor (*K*) of *H. leucisculus* and the concentrations of (a) total nitrogen (TN) and (b) total phosphorus (TP). The solid lines represent the results of logarithmic regression between the *K* and water quality variables, while the dashed lines indicate the 95% confidence intervals of the fitted models. The coefficient of determination ($R^{2}$) reflects the goodness of fit for the logarithmic regression models, while the *P* values indicate the statistical significance of the relationship, and *N* represents the number of sampling sites.

**FIGURE 6:**
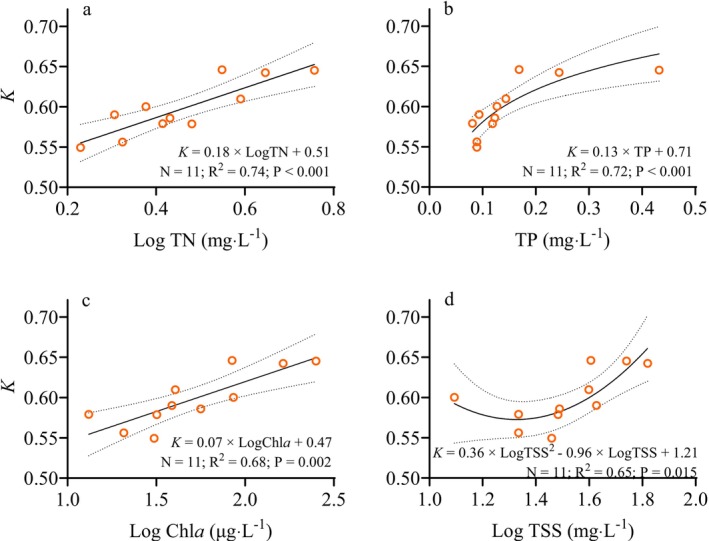
Relationship between the condition factor (*K*) of *T. swinhonis* and the concentrations of (a) total nitrogen (TN), (b) total phosphorus (TP), (c) chlorophyll‐*a* (Chl*a*), and (d) total suspended solids (TSS). The solid lines represent the results of linear regression between the *K* and water quality variables, while the dashed lines indicate the 95% confidence intervals of the fitted models. The coefficient of determination ($R^{2}$) reflects the goodness of fit for the linear regression models, while the *P* values indicate the statistical significance of the relationship, and *N* represents the number of sampling sites.

On the basis of the results of the GAM and GLM analyses (Table S2), the *K* value of *H. leucisculus* exhibited significant positive quadratic correlations with the concentrations of TN and TP, respectively (Figure 5). The *K* value of *T. swinhonis* increased significantly linearly with the concentrations of both TN and Chl*a* (Figure 6a,c). Moreover, *T. swinhonis K* increased significantly in a linear relationship with TP (Figure 6b) and in a quadratic relationship with TSS concentrations (Figure 6d).

**FIGURE 7:**
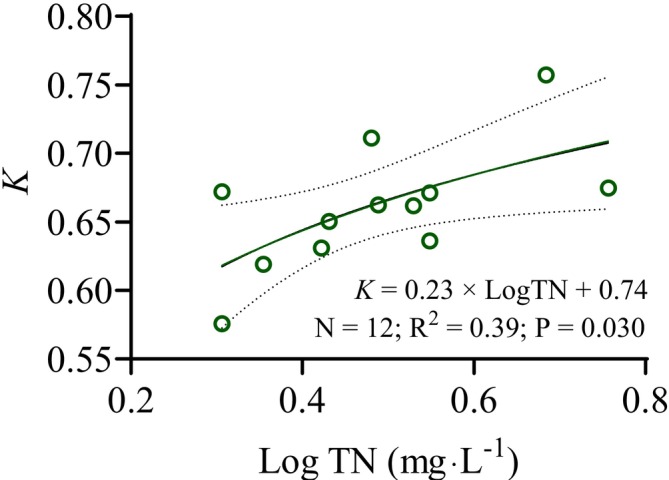
Relationship between the condition factor (*K*) of *Culter* spp. and the concentrations of total nitrogen (TN). The solid line represents the results of logarithmic regression between the *K* and TN, while the dashed line indicates the 95% confidence intervals of the fitted model. The coefficient of determination ($R^{2}$) reflects the goodness of fit for the logarithmic regression model, the *P* indicates the statistical significance of the relationship, and *N* represents the number of sampling sites.

The *K* values of *C. gibelio* and *P. simoni* showed no significant correlations with the measured water quality variables based on GAM and GLM results (Table S2). However, the *K* value of *Culter* spp. was positively correlated with TN concentrations and followed a logarithmic function (Figure 7 and Table S2).

## 4 Discussion

Our study revealed species‐specific responses in fish health status to nutrient enrichment across sampling sites. The condition factor (*K*) of both omni‐planktivorous *H. leucisculus* and zooplanktivorous *T. swinhonis* increased significantly with nutrient enrichment, aligning with our research hypothesis. However, the *K* values of the omni‐benthivorous *C. gibelio* and *P. simoni* remained largely unchanged, contradicting the hypothesis predicting an increase in omnivorous fish *K* alongside nutrient levels. Intriguingly, the *K* value of piscivorous *Culter* spp. increased significantly with TN, directly opposing the anticipated negative relationship between piscivores and nutrient enrichment.

The better health status of omni‐planktivorous *H. leucisculus* and zooplanktivorous *T. swinhonis* in nutrient‐enriched sites may be associated with the enhanced availability of food resources. In our study, phytoplankton biomass increased linearly with nutrient concentrations, which was in consistent with previous studies (Zou et al. 2020; Yu et al. 2021). Although we did not directly evaluate the relationship between changes in zooplankton biomass and nutrient concentrations, zooplankton biomass may increase significantly with both algal biomass and nutrient concentrations, as observed in Lake Taihu (Jia et al. 2017). The small‐sized zooplanktivorous *T. swinhonis* fed primarily on zooplankton (Yu et al. 2016). The omni‐planktivorous *H. leucisculus* has been observed to feed on both zooplankton and submerged macrophytes in macrophyte‐dominated restored lakes (Yu et al. 2016). However, we did not find high coverage of submerged macrophytes in our field samplings. Therefore, the enhanced food availability in nutrient‐rich rivers may sufficiently support the growth of these small fish, resulting in higher condition factors for both *H. leucisculus* and *T. swinhonis* in our study. In addition, the potential predation pressure exerted by piscivorous fish may be low in nutrient‐rich rivers, as the biomass of piscivorous fish has been found to decline significantly with nutrient enrichment in both subtropical and temperate freshwater ecosystems (Jeppesen et al. 2000; Yu et al. 2021). Furthermore, in our study, water transparency was low at nutrient‐enriched sites, which could reduce the foraging efficiency of visually oriented predators. (e.g., *Culter* spp.) (Jonsson et al. 2013). This, in turn, may indirectly reduce the predation risk faced by *H. leucisculus* and *T. swinhonis* from piscivorous fish, thereby potentially providing them with more favorable environments and leading to an increase in their condition factors.

The positive allometric growth of *H. leucisculus* in the present study aligns with findings from previous studies in freshwater lakes (Patimar et al. 2008; Wang et al. 2012), whereas it contrasts with the negative allometric growth documented for Dalai Lake and Luhun Reservoir (Liu et al. 2016). *T. swinhonis* exhibited isometric growth, which was inconsistent with both the positive allometric growth reported in Kuilei Lake and Tian'e‐zhou Oxbow Lake and the negative allometric growth observed in Biandan Lake and Shengjin Lake (Wang et al. 2012; Dong et al. 2019). Such discrepancies may arise from variations in sample size, body size structure, and sampling seasons across studies, as these factors have been reported to influence fish growth patterns (Hossain et al. 2012; Quang 2016; Zuev et al. 2017). Thus, these methodological differences may partly explain the observed regional and temporal variations in fish allometric growth observed in previous studies.

The omnivorous benthic‐feeding *C. gibelio* and *P. simoni* primarily consume detritus, benthic invertebrates, and macrophytes (Yu et al. 2016; Razlutskij et al. 2021). Here, correlations between the *K* and water quality variables were observed, though none reached statistical significance. Macroinvertebrate abundance increased with nutrient enrichment (Zhang et al. 2019), while submerged macrophytes coverage declined with elevated TP concentration (Sondergaard et al. 2022). Collectively, these findings indicate that the condition factors of both *C. gibelio* and *P. simoni* in our study were not significantly affected by the measured water quality variables, demonstrating their ability to adapt to fluctuating resource availability while maintaining healthy conditions. *C. gibelio* exhibited positive allometric growth in the present study, contrasting with reported isometric growth in Zhimai River and negative allometric growth in Ili River (Sui et al. 2015; Wang et al. 2015). *P. simoni* displayed isometric growth, differing from the negative allometric growth reported in Jingjiang River basin during 2002–2009 (Cheng et al. 2012), but consistent with isometric growth recorded in the same basin during 2012–2013 (Guo et al. 2017). Furthermore, substantial standard deviations and wide size ranges within the sampled fish populations may influence the precision and statistical significance of the allometric coefficient (*b*), with *b*‐value estimates potentially biased toward sites yielding higher individual counts.

The piscivorous *Culter* species primarily consume small fish and shrimp (Chen et al. 2009; Wang et al. 2019, 2022). Biomass of small fish species (e.g., *H. leucisculus* and *T. swinhonis* ) increased significantly with nutrient enrichment in shallow freshwaters (Sorensen et al. 2011; Yu et al. 2021). Consistent with this pattern, in the present study, we also caught more small‐sized fish in nutrient‐enriched sites (unpublished data). Consequently, the relatively elevated resource availability for *Culter* species likely accounts for the observed positive relationship between nutrient enrichment and their *K* in our study (Figure 7). Furthermore, most piscivores are visual predators, and their foraging efficiency is closely linked to water transparency. For instance, the foraging success of mandarin fish ( *Siniperca chuatsi* ) was substantially impaired under low‐light conditions (the algal‐induced turbidity over 80 NTU) (Li et al. 2013). In the present study, the maximum recorded NTU value (estimated using an empirical conversion from TSS (Saranga et al. 2021)) remained below this critical threshold. This may partly explain the absence of a statistically significant relationship between the *K* values of *Culter* spp. and TSS. Moreover, the concurrent increase in small fish availability associated with rising TN concentrations, which expanded the food source for *Culter* spp., may have partially offset potential negative impacts linked to elevated TSS levels (Jacobsen et al. 2014). The lack of statistical significance may also be associated with the constraint of limited sample size.

Our findings revealed that elevated nutrient levels correlated with higher or relatively stable condition factors in certain dominant species (e.g., *H. leucisculus* , *T. swinhonis* and *Culter* spp.), suggesting potentially “favorable” or “neutral” impacts of eutrophication at the individual level. However, such responses likely remain confined to a limited number of tolerant or opportunistic species. Yet, extensive evidence demonstrates that eutrophication triggers substantial declines in fish species diversity, simplification of community structure, and ecological niche compression (Jeppesen et al. 2003; Cheng et al. 2014; Horka et al. 2023), as well as food‐web simplification and reduced trophic transfer efficiency (Dickman et al. 2008; Xu et al. 2016). Consequently, these species‐specific outcomes must be interpreted not as indicators of ecosystem health, but as clear sighs of its degradation. The very success of these few species precisely signals the broader ecological imbalance inflicted by eutrophication. However, our study has several limitations. Firstly, sampling was conducted only in summer, a period with relatively high primary productivity. Seasonal variation may influence resource availability and fish condition (Nguyen et al. 2023), and therefore, the observed patterns may not fully represent fish health during other seasons. Secondly, no within‐site replication was performed in our study, and individual number for some species (excluding *H. leucisculus* and *T. swinhonis* ) were relatively low, limiting causal inference. Finally, we did not conduct direct measurements of fish diet composition, food resource availability, or predation pressure from piscivorous fish, instead of citing published literature. Therefore, our findings mainly reflect spatial relationships between condition factor and water quality variables, with limited evidence for causal links. Future research should integrate seasonal sampling, increased replication, and direct quantification of trophic interactions, such as gut content analysis, to elucidate the mechanisms driving fish condition in eutrophic systems.

## 5 Conclusion

We observed species‐specific responses in fish growth condition (*K*) across different feeding groups to nutrient enrichment within subtropical polder rivers. The *K* values of omni‐planktivores *H. leucisculus* , zooplanktivorous *T. swinhonis* , and piscivorous *Culter* spp. rose significantly with increasing nutrient levels. Conversely, the *K* values of the omnivorous *C. gibelio* and *P. simoni* remained largely unaffected by the water quality variables in our study. Crucially, it is vital to emphasize that *K* specifically reflects the growth status of dominant fish within eutrophic ecosystems and is unsuitable for assessing eutrophication's broader impacts on community structure or diversity.

## Author Contributions

**Jianwen Li:** data curation (lead), formal analysis (lead), investigation (equal), writing – original draft (lead), writing – review and editing (equal). **Zhenmei Lin:** data curation (supporting), investigation (supporting), writing – review and editing (supporting). **Jinlei Yu:** conceptualization (equal), investigation (equal), project administration (equal), supervision (equal), writing – review and editing (equal). **Zhigang Mao:** project administration (equal), writing – review and editing (equal). **Shi Fu:** data curation (supporting), investigation (supporting), writing – review and editing (supporting). **Kun Xu:** data curation (equal), investigation (equal), writing – review and editing (equal). **Chan Li:** data curation (supporting), investigation (supporting), writing – review and editing (equal). **Junfeng Gao:** funding acquisition (equal), writing – review and editing (equal). **Kuanyi Li:** conceptualization (equal), project administration (equal), writing – review and editing (equal). **Zhengwen Liu:** conceptualization (equal), writing – review and editing (equal).

## Funding

This research was funded by the National Key Research and Development Program of China (2023YFC3208705), National Natural Science Foundation of China (42277067), Jiangsu Provincial Science and Technology Planning Project (No. BK20231516), and State Key Laboratory of Lake and Watershed Science for Water Security (NKL2023‐KP02).

## Ethics Statement

All procedures involving the handling and sampling of fish in our study were reviewed and approved by the Animal Ethics Committee of the Nanjing Institute of Geography and Limnology, Chinese Academy of Sciences (Protocol/Approval Number: [NIGLAS2023AEC003]). All methods were performed in accordance with the relevant guidelines and regulations.

## Conflicts of Interest

The authors declare no conflicts of interest.

## Supporting information


**Table S1:** GPS information of the sampling sites for fish and water quality in rivers of Lake Chaohu Basin in Hefei, Anhui Province.
**Table S2:** Generalized additive model (GAM) analysis of relationships between fish health condition factor (*K*) and water quality variables. Water quality variables: total nitrogen (TN, mg・L^−1^), total phosphorus (TP, mg・L^−1^), chlorophyll‐*a* concentration in phytoplankton (Chl*a*, μg・L^−1^), and total suspended solids (TSS, mg・L^−1^). Fish health condition traits: condition factor (*K*).

**Data S1:** ece373353‐sup‐0002‐DataS1.xlsx.

**Data S2:** ece373353‐sup‐0003‐DataS2.docx.

## Data Availability

All the required data are uploaded in Supporting Information. The data and code are accessible to readers upon publication.
