# Supplementary material for: Species‐Specific Response of Fish Health Condition to Nutrient Enrichment in Subtropical Rivers
Source: Ecol Evol. 2026 Apr 2;16(4):e73353. doi: 10.1002/ece3.73353 (PMC13045326; doi:10.1002/ece3.73353)
Supplement: Supplementary file 1 — Table S1: GPS information of the sampling sites for fish and water quality in rivers of Lake Chaohu Basin in Hefei, Anhui Province. Table S2: Generalized additive model (GAM) analysis of relationships between fish health condition factor (K) and water quality variables. Water quality variables: total nitrogen (TN, mg・L−1), total phosphorus (TP, mg・L−1), chlorophyll‐a concentration in phytoplankton (Chla, μg・L−1), and total suspended solids (TSS, mg・L−1). Fish health condition traits: condition factor (K). [file ECE3-16-e73353-s001.docx]

**Species-specific response of fish health condition to nutrient enrichment in subtropical rivers**

Jianwen Li^1, 2^, Zhenmei Lin^1, 2^, Jinlei Yu^1, 2, 3*^, Zhigang Mao^1, 2*^, Shi Fu^1, 2^, Kun Xu^1, 2^, Chan Li^1^, Junfeng Gao^1*^, Kuanyi Li^1^ and Zhengwen Liu^1, 2, 3, 4^

^1^State Key Laboratory of Lake and Watershed Science for Water Security, Nanjing Institute of Geography and Limnology, Chinese Academy of Sciences, Nanjing 211135, China

^2^University of Chinese Academy of Sciences, Beijing 100049, China

^3^Sino-Danish Centre for Education and Research , University of Chinese Academy of Sciences, Beijing 100190, China

^4^Department of Ecology, Jinan University, Guangzhou 510630, China

Correspondence: Jinlei Yu (jlyu@niglas.ac.cn) | Zhigang Mao (zgmao@niglas.ac.cn) | Junfeng Gao (gaojunf@niglas.ac.cn)

**Table S1**: GPS information of the sampling sites for fish and water quality in rivers of Lake Chaohu Basin in Hefei, Anhui Province. **Table S2**: Generalized additive model (GAM) analysis of relationships between fish health condition factor (*K*) and water quality variables. Water quality variables: total nitrogen (TN, mg・L^-1^), total phosphorus (TP, mg・L^-1^) chlorophyll-*a* concentration in phytoplankton (Chl*a*, μg・L^-1^) and total suspended solids (TSS, mg・L^-1^). Fish health condition traits: condition factor (*K*).

**TABLE S1** GPS information of the sampling sites for fish and water quality.

| **Sites** | **Lon** | **Lat** | **Sites** | **Lon** | **Lat** |
| --- | --- | --- | --- | --- | --- |
| **1** | 117.340119 | 31.838615 | **21** | 117.077735 | 31.554650 |
| **2** | 117.458922 | 31.894364 | **22** | 117.091259 | 31.338645 |
| **3** | 117.302180 | 31.852001 | **23** | 117.310202 | 31.484602 |
| **4** | 117.444557 | 31.753699 | **24** | 117.270486 | 31.429288 |
| **5** | 117.389645 | 31.837217 | **25** | 117.274312 | 31.353858 |
| **6** | 117.455234 | 31.822717 | **26** | 117.232680 | 31.423492 |
| **7** | 117.386511 | 31.765704 | **27** | 117.512895 | 31.330101 |
| **8** | 117.223768 | 31.679840 | **28** | 117.433949 | 31.155821 |
| **9** | 117.083800 | 31.798000 | **29** | 117.427735 | 31.029574 |
| **10** | 117.223195 | 31.635621 | **30** | 117.583690 | 31.313765 |
| **11** | 117.180157 | 31.716889 | **31** | 117.464653 | 31.389061 |
| **12** | 117.305737 | 31.731405 | **32** | 117.472183 | 31.226982 |
| **13** | 117.251327 | 31.617777 | **33** | 117.303288 | 31.227375 |
| **14** | 117.163970 | 31.586414 | **34** | 117.322238 | 31.075391 |
| **15** | 117.165509 | 31.550045 | **35** | 117.785243 | 31.732249 |
| **16** | 116.853787 | 31.530218 | **36** | 117.839413 | 31.704468 |
| **17** | 117.253091 | 31.504569 | **37** | 117.760809 | 31.764074 |
| **18** | 117.228893 | 31.529670 | **38** | 117.857384 | 31.762475 |
| **19** | 116.951630 | 31.567032 | **39** | 117.635252 | 31.697475 |
| **20** | 116.929410 | 31.625536 | **40** | 117.666269 | 31.696430 |

**TABLE S2** Generalized additive model (GAM) analysis of relationships between fish health condition

factor (*K*) and water quality variables.

| **Species** | **Term** | **edf** | **F** | **P** | **AIC** | $\text{R}_{\text{adj}}^{\text{2}}$ |
| --- | --- | --- | --- | --- | --- | --- |
| ***Hemiculter leucisculus*** | TN | 1.71 | 10.15 | **0.001** | -77.24 | 0.54 |
|  | TP | 1.75 | 6.88 | **0.014** | -71.23 | 0.37 |
|  | Chl*a* | 1.68 | 1.62 | 0.292 | -64.18 | 0.09 |
|  | TSS | 1.00 | 0.67 | 0.425 | -62.62 | -0.02 |
| ***Toxabramis swinhonis*** | TN | 1.00 | 25.57 | **0.001** | -52.91 | 0.71 |
|  | TP | 1.92 | 17.68 | **0.001** | -55.48 | 0.78 |
|  | Chl*a* | 1.00 | 19.27 | **0.002** | -50.69 | 0.65 |
|  | TSS | 1.86 | 8.00 | **0.017** | -48.05 | 0.57 |
| ***Carassius gibelio*** | TN | 1.00 | 0.39 | 0.553 | -6.05 | -0.09 |
|  | TP | 1.85 | 3.16 | 0.139 | -10.67 | 0.42 |
|  | Chl*a* | 1.00 | 0.07 | 0.794 | -5.64 | -0.15 |
|  | TSS | 1.00 | 0.25 | 0.635 | -5.87 | -0.12 |
| ***Pseudobrama simoni*** | TN | 1.00 | 0.01 | 0.928 | -7.93 | -0.20 |
|  | TP | 1.00 | 0.01 | 0.943 | -7.93 | -0.20 |
|  | Chl*a* | 1.00 | 0.02 | 0.887 | -7.95 | -0.19 |
|  | TSS | 1.00 | 1.87 | 0.230 | -10.14 | 0.13 |
| ***Culter* spp. (combined *Culter mongolicus* and *Culter alburnus*)** | TN | 1.00 | 6.21 | **0.032** | -40.90 | 0.32 |
|  | TP | 1.00 | 0.01 | 0.939 | -35.11 | -0.10 |
|  | Chl*a* | 1.85 | 3.16 | 0.079 | -40.80 | 0.35 |
|  | TSS | 1.00 | 1.07 | 0.326 | -36.32 | 0.01 |
