# Supplementary material for: Species‐Specific Response of Fish Health Condition to Nutrient Enrichment in Subtropical Rivers
Source: Ecol Evol. 2026 Apr 2;16(4):e73353. doi: 10.1002/ece3.73353 (PMC13045326; doi:10.1002/ece3.73353)
Supplement: Supplementary file 3 — Data S2: ece373353‐sup‐0003‐DataS2.docx. [file ECE3-16-e73353-s002.docx]

**Species-specific response of fish health condition to nutrient enrichment in subtropical rivers**

Jianwen Li^1, 2^, Zhenmei Lin^1, 2^, Jinlei Yu^1, 2, 3*^, Zhigang Mao^1, 2*^, Shi Fu^1, 2^, Kun Xu^1, 2^, Chan Li^1^, Junfeng Gao^1*^, Kuanyi Li^1^ and Zhengwen Liu^1, 2, 3, 4^

^1^State Key Laboratory of Lake and Watershed Science for Water Security, Nanjing Institute of Geography and Limnology (NIGLAS), Chinese Academy of Sciences, Nanjing 211135, China

^2^University of Chinese Academy of Sciences, Beijing 100049, China

^3^Sino-Danish Centre for Education and Research (SDC), University of Chinese Academy of Sciences, Beijing 100190, China

^4^Department of Ecology, Jinan University, Guangzhou 510630, China

Correspondence: Jinlei Yu (jlyu@niglas.ac.cn) | Zhigang Mao (zgmao@niglas.ac.cn) | Junfeng Gao [(gaojunf@niglas.ac.cn)](mailto:(gaojunf@niglas.ac.cn))

The data required for the manuscript has been compiled in the Excel file named "Data", with each worksheet labeled according to the specific content it contains. Below are the R codes utilized in the manuscript's analyses. You can easily locate the corresponding data within the "data" file based on the functional descriptions provided for each section of the code. To facilitate efficient and accurate reproduction of the results, we recommend creating a new Excel file where you can paste the data required for each segment of code before executing them in *RStudio*.

**1.** Analyze the Length-Weight relationships (LWR) of 6 fish species and conduct visualization.

library(readxl)

library(ggplot2)

library(car)

data <- read_excel(file.choose(), sheet = "Sheet1")

n <- nrow(data)

model <- lm(log(W) ~ log(L), data = data)

summary(model)

a <- exp(coef(model)[1])

b <- coef(model)[2]

p_value <- round(summary(model)$coefficients[2,4],3)

r_squared <- round(summary(model)$r.squared,2)

a_formatted <- formatC(a, format = "fg", digits =2)

b_formatted <- formatC(b, format = "fg", digits = 4)

cat("result：\n")

cat("a =", a, "\n")

cat("b =", b, "\n")

cat("P value:", p_value, "\n")

cat("R2:", r_squared, "\n")

conf_interval <- confint(model, level = 0.95)

b_lower <- conf_interval["log(L)", "2.5 %"]

b_upper <- conf_interval["log(L)", "97.5 %"]

cat(" 95% confidence intervals of b：[", formatC(b_lower, format = "fg", digits =5),

",", formatC(b_upper, format = "fg", digits =5), "]\n")

hypothesis_test <- linearHypothesis(model, "log(L) = 3")

p_value_b_vs_3 <- hypothesis_test$`Pr(>F)`[2]

cat("difference of 3:", round(p_value_b_vs_3, 4), "\n")

ggplot(data, aes(x =L, y = W)) +

geom_point(color = "blue", size =5,shape=1) +

stat_smooth(method = "nls",formula = y ~ a * x^b, method. args = list(start = list(a = 1, b = 1)), se = FALSE, color = "grey50", linewidth=1.2) +

xlab(expression(bold(L)~ plain("(") *cm* plain(")"))) +

ylab(expression(bold(W)~plain("(") *g * plain(")"))) +

theme_classic() +

theme(

plot.title = element_text(color = "black", size = 35, family = "serif", hjust = 0.5),

axis.title.x = element_blank(),

axis.title.y = element_blank(),

axis.ticks.length = unit(0.2, "cm"),

axis.text = element_text(size =25),

panel.border = element_rect(color="black",fill=NA,linewidth = 0.7))+

scale_x_continuous(breaks = seq(from =5, to =28, by =5), limits = c(5,28), expand = c(0.01, 0.01)) +

scale_y_continuous(breaks = seq(from = 0, to =68, by =10), limits = c(0,68), expand = c(0.01, 0.01)) +

annotate("text",x=15,y=5,label=bquote(W==.(a_formatted)* L^.(b_formatted)),size =12,hjust = 0,family = "serif")+

annotate("text", x =13.7, y =2, label = bquote(N == .(n) * " " ~ R^2 == .(r_squared) * " " ~ P == .(p_value)), size = 12, hjust = 0)+

annotate("text", x =5.3, y =65, label = "(a)", fontface = "bold", family = "serif", size =12, hjust = 0)+

annotate("text", x =6, y =65, label = "H.leucisculus", fontface = "bold.italic", family = "serif", size =12, hjust = 0)

1. Correlation analysis encompassing two aspects: inter-variable water quality correlations, and correlations between condition factor (*K*) and water quality variables.

**Note**: In the code, "TN", "TP", "Chla", and "TSS" denote preprocessed water quality variables described in the **Statistical analysis** section; the "log" notation is omitted for simplicity, **hereafter**.

library(GGally)

library(ggplot2)

library(readxl)

library(grid)

dt1 <- read_excel(file.choose(), sheet = "Sheet1")

dt <- dt1[, c("TN", "TP", "Chla", "TSS")]

#adding "*K*" if analysis the correlation between K and water quality variables

my_cor <- function(data, mapping, ...) {

x <- eval_data_col(data, mapping$x)

y <- eval_data_col(data, mapping$y)

corr <- cor.test(x, y,method=c("spearman"))

r <- round(corr$estimate, 2)

p <- corr$p.value

stars <- ifelse(p < 0.001, "***",

ifelse(p < 0.01, "**",

ifelse(p < 0.05, "*", "")))

GGally::ggally_text(

label = paste0("r = ", r, "\n", stars),

mapping = aes(),

size=12,

...

)

}

p <- ggpairs(

dt,

title = "",

upper = list(continuous = my_cor,color="black"),

lower = list(

continuous = wrap(

"smooth",

size =1,

shape = 1,

color = "green",

fill = "white",

alpha = 1,

stroke = 0.8

)

),

diag = list(continuous = wrap("densityDiag", alpha = 0.6, color="green", fill =NA))

)

p <- p + theme_bw(base_size =18) +

theme( panel.grid = element_blank(),

plot.title = element_text(hjust = 0.5, size = 16),

strip.text = element_text(size = 16),

panel.border = element_rect(color = "black", linewidth = 0.5))

print(p)

**3.** Heatmap visualization of correlations between the condition factor (*K*) of fish with different feeding habits and water quality variables. **Note:** The correlations between *K* and water quality variables have been calculated and stored in the "Heatmap" Excel file.

library(readxl)

library(ggplot2)

library(pheatmap)

library(reshape2)

library(RColorBrewer)

library(gplots)

data <- read_excel(file.choose(), sheet = "Sheet1")

r_data <- data[, c("Variables",

"Sharpbelly r", "Sharpbelly P",

"Sijiao r", "Sijiao P",

"Prussian carp r", "Prussian carp P",

"Sibian r", "Sibian P",

"Culter r", "Culter P")]

r_data <- as.data.frame(r_data)

rownames(r_data) <- r_data$Variables

r_data$Variables <- NULL

r_matrix <- as.matrix(r_data[, c(1, 3, 5, 7, 9)])

p_matrix <- as.matrix(r_data[, c(2, 4, 6, 8, 10)])

colnames(r_matrix) <- c("Sharpbelly", "Sijiao", "Prussian carp", "Sibian", "Culter")

colnames(p_matrix) <- colnames(r_matrix)

rownames(p_matrix) <- rownames(r_matrix)

r_matrix[is.na(r_matrix)] <- 0

p_matrix[is.na(p_matrix)] <- 1

significance_marks <- function(p) {

if (p < 0.001) return("***")

if (p < 0.01) return("**")

if (p < 0.05) return("*")

return("")

}

sign_matrix <- matrix(sapply(p_matrix, significance_marks),

nrow = nrow(p_matrix), ncol = ncol(p_matrix))

rownames(sign_matrix) <- rownames(p_matrix)

colnames(sign_matrix) <- colnames(p_matrix)

display_matrix <- matrix(formatC(r_matrix, format = "f", digits = 2),

nrow = nrow(r_matrix), ncol = ncol(r_matrix))

display_matrix_with_sign <- matrix(paste0(display_matrix, sign_matrix),

nrow = nrow(r_matrix),

ncol = ncol(r_matrix))

rownames(display_matrix_with_sign) <- rownames(r_matrix)

colnames(display_matrix_with_sign) <- colnames(r_matrix)

red_gradient <- colorRampPalette(c("#d7302770", "#f7f7f7"))(40)

blue_gradient <- colorRampPalette(c("#f7f7f7", "#4575b4"))(100)

color_palette <- c(red_gradient, blue_gradient)

color_breaks <- seq(-0.2, 1.0, length.out = 121)

pdf("D:/R/heatmap.pdf", width = 14, height = 10)

pheatmap(r_matrix,

cluster_rows = FALSE,

cluster_cols = FALSE,

display_numbers = display_matrix_with_sign,

color = color_palette,

breaks = color_breaks,

fontsize = 20,

fontsize_row = 24,

fontsize_col = 24,

show_rownames = TRUE,

show_colnames = TRUE,

angle_col = 45,

border_color = "white",

tree_row_height = 0,

tree_col_width = 0,

number_format = function(x) { sprintf("%.3f", as.numeric(x)) })

dev.off()

**4.** Generalized Additive Models (GAM) were constructed to explore the relationships between the condition factor (*K*) of fish with different feeding habits and water quality variables.

library(readxl)

library(mgcv)

library(DHARMa)

data <- read_excel(file.choose(), sheet="Sheet1")

var_combinations <- list(c("TN"), c("TP"), c("Chla"), c("TSS"))

dependent_vars <- c("K")

results <- data.frame(

Dependent_Var = character(),

Model = character(),

Term = character(),

edf = numeric(),

Ref_df = numeric(),

F_value = numeric(),

Pr_gt_F = numeric(),

Residual_Deviance = numeric(),

AIC = numeric(),

Adj_R_Squared = numeric(),

Explained_Deviance = numeric(),

k_index = numeric(),

k_p_value = numeric(),

Shapiro_W_p = numeric(),

DHARMa_BP_P = numeric(),

ACF_Max = numeric(),

stringsAsFactors = FALSE

)

for(dep_var in dependent_vars){

for(vars in var_combinations){

smooth_terms <- paste0("s(", vars, ", k=3)", collapse = " + ")

formula <- as.formula(paste(dep_var, "~", smooth_terms))

model_name <- paste("GAM", dep_var, paste(vars, collapse="_"), sep="_")

model <- gam(formula, data=data, family=gaussian())

model_summary <- summary(model)

gam_chk <- gam.check(model, print=FALSE)

k_index <- if(!is.null(gam_chk$k.check)) gam_chk$k.check[1,"k-index"] else NA

k_p_value <- if(!is.null(gam_chk$k.check)) gam_chk$k.check[1,"p-value"] else NA

res <- residuals(model, type="pearson")

fitted_vals <- fitted(model)

shapiro_p <- shapiro.test(res)$p.value

sim_res <- simulateResiduals(fittedModel = model, n = 1000)

dharma_bp_p <- testDispersion(sim_res)$p.value

acf_vals <- acf(res, plot=FALSE)$acf[-1]

acf_max <- max(abs(acf_vals))

smooth_table <- model_summary$s.table

residual_dev <- model$deviance

aic_val <- AIC(model)

adj_r2 <- model_summary$r.sq

dev_exp <- model_summary$dev.expl

if(!is.null(smooth_table) && nrow(smooth_table) > 0){

for(j in 1:nrow(smooth_table)){

results <- rbind(results, data.frame(

Dependent_Var=dep_var,

Model=model_name,

Term=rownames(smooth_table)[j],

edf=smooth_table[j,"edf"],

Ref_df=smooth_table[j,"Ref.df"],

F_value=smooth_table[j,"F"],

Pr_gt_F=smooth_table[j,"p-value"],

Residual_Deviance=residual_dev,

AIC=aic_val,

Adj_R_Squared=adj_r2,

Explained_Deviance=dev_exp,

k_index=k_index,

k_p_value=k_p_value,

Shapiro_W_p=shapiro_p,

DHARMa_BP_P=dharma_bp_p,

ACF_Max=acf_max,

stringsAsFactors=FALSE

))

}

} else {

results <- rbind(results, data.frame(

Dependent_Var=dep_var,

Model=model_name,

Term=NA,

edf=NA,

Ref_df=NA,

F_value=NA,

Pr_gt_F=NA,

Residual_Deviance=residual_dev,

AIC=aic_val,

Adj_R_Squared=adj_r2,

Explained_Deviance=dev_exp,

k_index=k_index,

k_p_value=k_p_value,

Shapiro_W_p=shapiro_p,

DHARMa_BP_P=dharma_bp_p,

ACF_Max=acf_max,

stringsAsFactors=FALSE

))

}

plot(fitted_vals, res,

xlab="Fitted values", ylab="Pearson residuals",

main=paste("Residuals vs Fitted:", model_name))

abline(h=0, lty=2)

acf(res, main=paste("ACF of Residuals:", model_name))

}

}

write.csv(results, "GAM_full_diagnostics_DHARMa.csv", row.names = FALSE)

**5.** Logarithmic regression analysis was conducted to model the relationship between each water quality variable and the condition factor (*K*). All relationships were evaluated using three different regression forms (e.g., logarithmic, quadratic, linear), and the optimal model for each variable-*K* pair was selected based on the Akaike Information Criterion (AIC) values.

library(readxl)

library(ggplot2)

# logarithmic regression

data <- read_excel(file.choose(), sheet = "Sheet1")

fit_log <- lm(K ~ log10(TN), data = data)

summary(fit_log)

cat("=== Log-linear regression model (K ~ log10(TN)) parameters ===\n")

cat("Intercept (a):", round(coef(fit_log)[1], 4), "\n")

cat("log10(TN) coefficient (b):", round(coef(fit_log)[2], 4), "\n")

cat("Coefficient of determination R²:", round(summary(fit_log)$r.squared, 4), "\n")

cat("AIC value:", round(AIC(fit_log), 4), "\n\n")

# quadratic regression

fit_quad <- lm(K ~ TN + I(TN^2), data = data)

summary(fit_quad)

cat("=== Quadratic regression model (K ~ TN + TN²) parameters ===\n")

cat("Intercept (a):", round(coef(fit_quad)[1], 4), "\n")

cat("TN linear term coefficient (b1):", round(coef(fit_quad)[2], 4), "\n")

cat("TN squared term coefficient (b2):", round(coef(fit_quad)[3], 4), "\n")

cat("Coefficient of determination R²:", round(summary(fit_quad)$r.squared, 4), "\n")

cat("AIC value:", round(AIC(fit_quad), 4), "\n\n")

# linear regression

fit<- lm(K ~ TN, data = data)

summary(fit)

cat("=== Linear regression model (K ~ TN) parameters ===\n")

cat("Intercept (a):", round(coef(fit)[1], 4), "\n")

cat("TN coefficient (b):", round(coef(fit)[2], 4), "\n")

cat("Coefficient of determination R²:", round(summary(fit)$r.squared, 4), "\n")

cat("AIC value:", round(AIC(fit), 4), "\n")

**6.** The following code exemplifies the visualization of linear regression between the condition factor (*K*) of sharpbelly (*Hemiculter leucisculus*) and total nitrogen (TN). The visualization approaches for other water quality variables and alternative regression forms (e.g., logarithmic, quadratic) follow this similar framework.

pred <- predict(fit, newdata = data, se.fit = TRUE)

data$fit <- pred$fit

data$upr <- pred$fit + 1.96 * pred$se.fit

data$lwr <- pred$fit - 1.96 * pred$se.fit

p <- ggplot(data, aes(x = TN, y = K)) +

geom_point(shape = 16, color = "grey0", size = 5, alpha = 0.5) +

geom_line(aes(y = fit), color = "black", linewidth = 1) +

geom_line(aes(y = upr), linewidth = 0.5, linetype = "dashed", color = "grey50") +

geom_line(aes(y = lwr), linewidth = 0.5, linetype = "dashed", color = "grey50") +

theme_classic() +

theme(

plot.title = element_text(color = "black", size = 35, family = "serif", hjust = 0.5),

axis.title.x = element_text(color = "black", size = 40, family = "serif"),

axis.title.y = element_text(color = "black", size = 40, family = "serif"),

axis.line.x = element_line(linewidth = 1, color = "black"),

axis.line.y = element_line(linewidth = 1, color = "black"),

axis.ticks.length = unit(0.2, "cm"),

axis.text = element_text(size = 38),

plot.margin = margin(0.1, 0.1, 0.1, 0.1, "cm")

) +

xlab(expression(bold(TN)~ plain("(") * m*g ~ L^{-1} * plain(")"))) +

ylab(expression(bolditalic(K))) +

scale_x_continuous(limits = c(0.2, 1.0), breaks = seq(from = 0.2, to = 1.0, by = 0.2)) +

scale_y_continuous(limits = c(0.60, 0.83), breaks = seq(from = 0.60, to = 0.83, by = 0.05))

print(p)
